# Adult-onset asthma morbidity and related economic costs in middle age due to intentional chronic absenteeism in high school: An epidemiologic study using the national longitudinal survey of youth 1979 data

**DOI:** 10.1371/journal.pone.0306451

**Published:** 2024-08-02

**Authors:** Zongqiang Liao, Neville Francis, Kevin Brooks

**Affiliations:** 1 Institute for Health Policy, Michigan State University, East Lansing, Michigan, United States of America; 2 Department of Economics, University of North Carolina at Chapel Hill, Chapel Hill, North Carolina, United States of America; University of Southern Queensland, AUSTRALIA

## Abstract

**Objective:**

Many authors examined the individual and societal impact of school absenteeism. Nevertheless, no empirical study has looked at the potential direct correlation between deliberate school absences and chronic illnesses in mid-adulthood. Our goal is to investigate any potential direct links between purposeful school absences and adult-onset asthma in middle age, as well as measure any associated costs of asthma.

**Methods:**

Data were sourced from the National Longitudinal Survey of Youth 1979, a nationally representative survey. The outcome measure was self-reported asthma in mid-adulthood. School records of absenteeism from grades nine through twelve were the key explanatory variables. Logistic regressions were performed with controls for demographic, economic and health variables. Predicted probabilities from the regressions were used to quantify costs of adult-onset asthma in middle age due to intentional high school absenteeism.

**Results:**

More years of chronic absenteeism in high school were associated with higher risk of adult-onset asthma in middle age. Four years of chronic absenteeism in high school during the late 1970s through the early 1980s could potentially have incurred between $817 million to $1 billion of asthma related costs in 2002, when these students were in their mid-adulthood. These potential asthma related costs due to high school absenteeism are sizeable considering that this high school cohort only accounted for six percent of the U.S. population.

**Conclusions:**

Reducing high school absenteeism could lower the incidence of adult-onset asthma in middle age, and its associated future economic burden.

## Introduction

Some people regularly missed school as teenagers, for good or bad reasons. Before 2016, the U.S. Department of Education defined chronic absenteeism (CA) as an excused or unexcused absence from school for 15 or more days during the school year (https://www2.ed.gov/datastory/chronicabsenteeism.html). In 2016, the Department of Education changed the definition of CA to include missing at least 10% of school days in a year. From 2013 to 2014, more than 3 million U.S. high school students, or 19% of U.S. high school students, met the definition of CA [1]. CA is often related to living conditions and other environmental factors [2]. However, some teens simply skipped school because they or their parents did not see the value in going to school.

Whether the absence was circumstantial or voluntary, the effects of CA can last a lifetime and negatively impact health in the long term, such as in middle age. The current literature has shown that CA leads to advert future health conditions in the long run through a series of steps. First, CA tends to have a negative impact on an individual’s academic performance, school and social participation, and health behaviors [3, 4]. CA in early childhood is a predictor of future CA in later grades, especially in high school. Furthermore, students with CA are at higher risk of dropping out of school and are much less likely to enrol in university [5–8]. As a result, CA is associated with a reduction in the quantity and quality of formal education obtained, which has been shown to impact future health [9–13]. One explanation was economic, as education is associated with income and career choices. Adults with lower levels of education are more likely to be unemployed, have lower incomes, and receive fewer benefits over their lifetime. Therefore, they are less likely to have fulfilling or successful careers in mid-adulthood and often exhibit unhealthy behaviors, psychological problems, increased inflammation, and decreased immune system function [14]. Another explanation was cognitive, with lower levels of education associated with lower health literacy and decision-making. Therefore, people with lower levels of education are more likely to engage in behaviors that jeopardize their current and future health, such as smoking and drinking [15, 16] and being overweight, all of which are directly related to certain chronic diseases such as asthma. Finally, frequent absences may indicate a lack of connection to school. Research has shown that attachment to school in teens is associated with improved mental health, reduced violent and suicidal behavior, and reduced substance use in adulthood [17, 18]. Individuals who start smoking or drinking at an early age tend to develop a lifelong addiction that can be extremely hard to quit. In summary, CA leads to lower educational and occupational fulfilment and is associated with worse economic, social, and health status in adulthood [19].

Asthma is one of the most common chronic diseases in the United States. According to the latest national asthma data from the Centers for Disease Control and Prevention (CDC) (https://www.cdc.gov/asthma/most_recent_national_asthma_data.htm), 8.0% of American adults had asthma in 2021. The American Lung Association Scientific and Medical Editorial Review Panel has shown the following factors that increase a person’s risk for developing asthma: family history, allergies, viral respiratory infections, occupational exposures, smoking, air pollution and obesity. Asthma morbidity and mortality are associated with exposure to allergens in the home and neighborhood environment. The more exposure an individual has to allergens, the more likely the individual is to develop asthma symptoms. Socioeconomic factors, especially income level, are known to significantly influence allergen exposure [20]. Low-income households tend to have more exposure to allergens than high-income households [21, 22]. The proportion of low-income households with elevated allergen levels was found to be five times that of the average U.S. household. The incidence of asthma was twice the US population average [23]. As a chronic disorder, individuals with asthma have incurred a substantial amount of economic burden on the society. The total costs of asthma include both direct health care expenditures and indirect costs, such as lost days at work or school because of morbidity and productivity loss from mortality [24].

**Fig 1:**
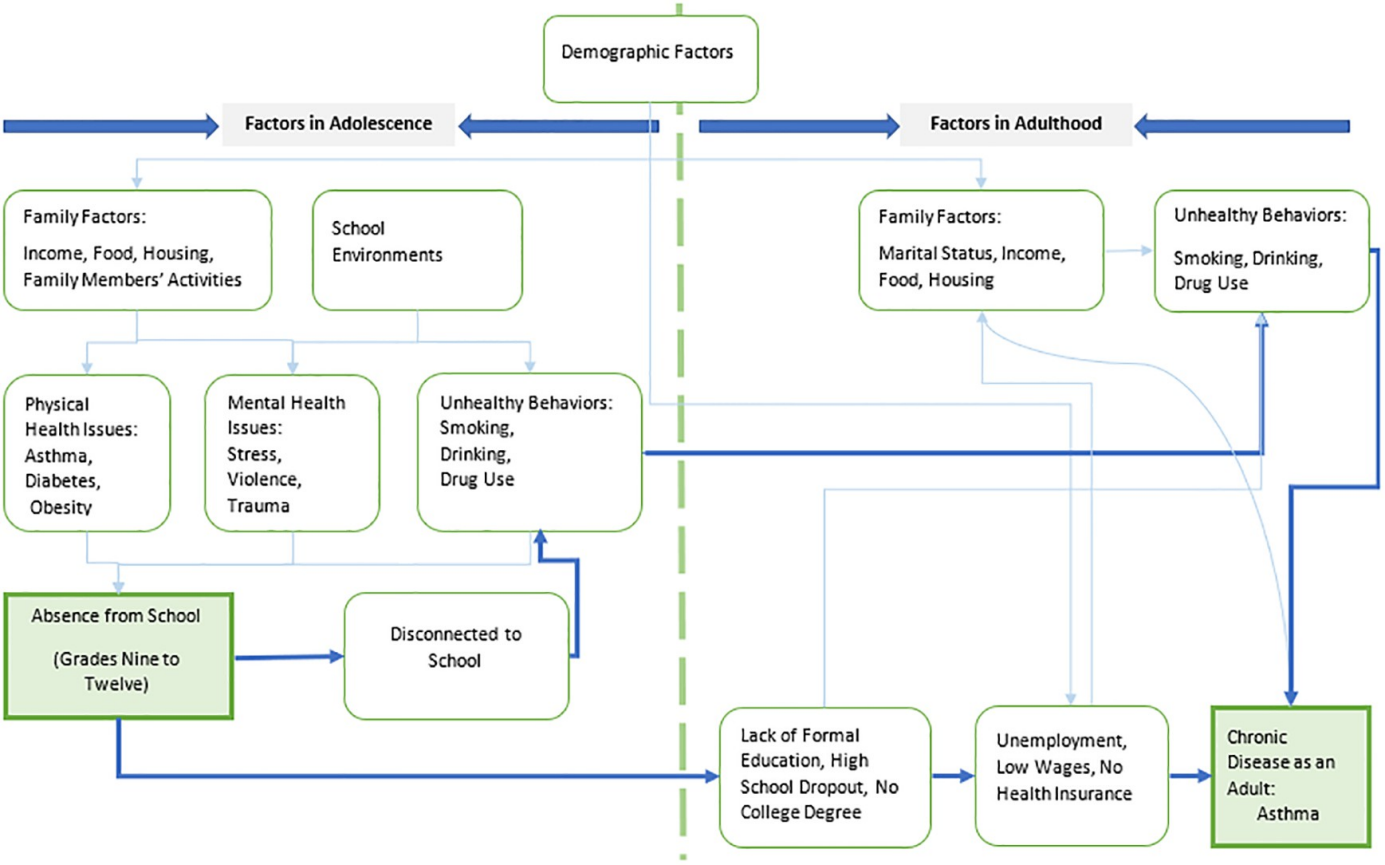
Conceptual pathway from adolescent school absenteeism to chronic disease in mid-adulthood and mediating factors.

In the studies reviewed so far, CA is not listed as a contributing factor for adult-onset asthma in middle age. Our study hypothesized that CA during high school is directly associated with long-term negative health outcomes (particularly adult-onset asthma) in mid-adulthood. Meanwhile, we also considered two other hypotheses in existing literature that the relationship between high school CA and having chronic disease in middle age through the steps: (i) smoking and drinking in the adolescent age as the results of low education level or disconnecting to school; (ii) low income due to school dropout. We developed a theoretical framework based on above reviewed research and our hypotheses (Fig 1). This framework included CA in high school, whether a person engaged in unhealthy behaviors such as smoking or drinking in the adolescence, and highest education levels and financial factors that may be limiting the living and working environment. In addition, we controlled for demographic factors and key risk factors such as gender, obesity and family history.

Our theoretical framework applies to nearly all chronic illnesses, including adult-onset asthma. According to recent research, mid-adulthood health can be impacted by CA through several different mechanisms. Academic progress can be hindered by CA, which leads to reduced educational attainment and a disconnection from the school system. It can also anticipate harmful health problems (like obesity) and habits (like smoking) throughout life, which are eventually linked to adult-onset asthma in middle age. Additionally, a lower level of education typically translates into future unemployment and lower income, which is linked to environmental allergen exposure and, ultimately, to adult-onset asthma. Regular school absences therefore cause a lifetime of regrettable yet avoidable effects for children, such as reduced adult salaries and worse health in the adulthood. However, there is not much empirical work on the direct impacts of missing school on one’s health status in mid-adulthood. In one noteworthy instance, absenteeism in childhood was documented to be a predictor of poor physical and behavioral health [14] in later ages. Similarly, a different study [25] found a link between children’s school absenteeism and mid-adulthood negative outcomes, such as alcohol abuse and mental health issues.

In contrast to earlier research of this type, the primary goal of this study is to investigate any possible direct correlation between non-childhood-health related school absences and adult-onset asthma in mid-adulthood. It is often known that health issues in young people, such as childhood asthma, can cause missed school days. Our study question, however, is the reverse. Is missing school linked to adult-onset asthma in middle age? Thus, for two reasons, this study did not include participants with underlying medical issues who were in their teens (before the age of 18): (i) We designed our primary explanatory measure of school absenteeism to account for deliberate absences from school; (ii) This exclusion potentially accounts for any reverse causality between childhood health and school absenteeism, as health status in mid-adulthood was the outcome variable (https://www.cdc.gov/asthma/asthma_stats/missing_days.htm).

In this paper, we used the National Longitudinal Survey of Youth 1979 (NLSY79) data to evaluate the relationship between intentional high school CA and adult-onset asthmatic outcome at 40 years of age, as well as monetizing its economic burden. We also allowed for two other linkages along with key risk factors for asthma. What we did in our analyses is, to our knowledge, unprecedented. Examining the effect of intentional high school absences on mid-adulthood health outcomes provides a more in-depth understanding of why reducing non-childhood-health related absences can inform future economic burden policies for adult-onset asthma.

## Methods

### Data and sample

We conducted secondary analyses using the cohort in the NLSY79, which started in 1979 and initially included 12,686 youths aged 14 to 22. The NLSY79 was originally designed by the Bureau of Labor Statistics to collect a profile of American youths’ school-to-work transition in the late 1970s and has been expanded to collect much broader contents over the past decades. The NLSY79 is a nationally representative, longitudinal study and with high response rates and frequent follow-ups. Most surveys were conducted on an annual or biennial basis. The NLSY79 data is de-identified and publicly available.

**Fig 2:**
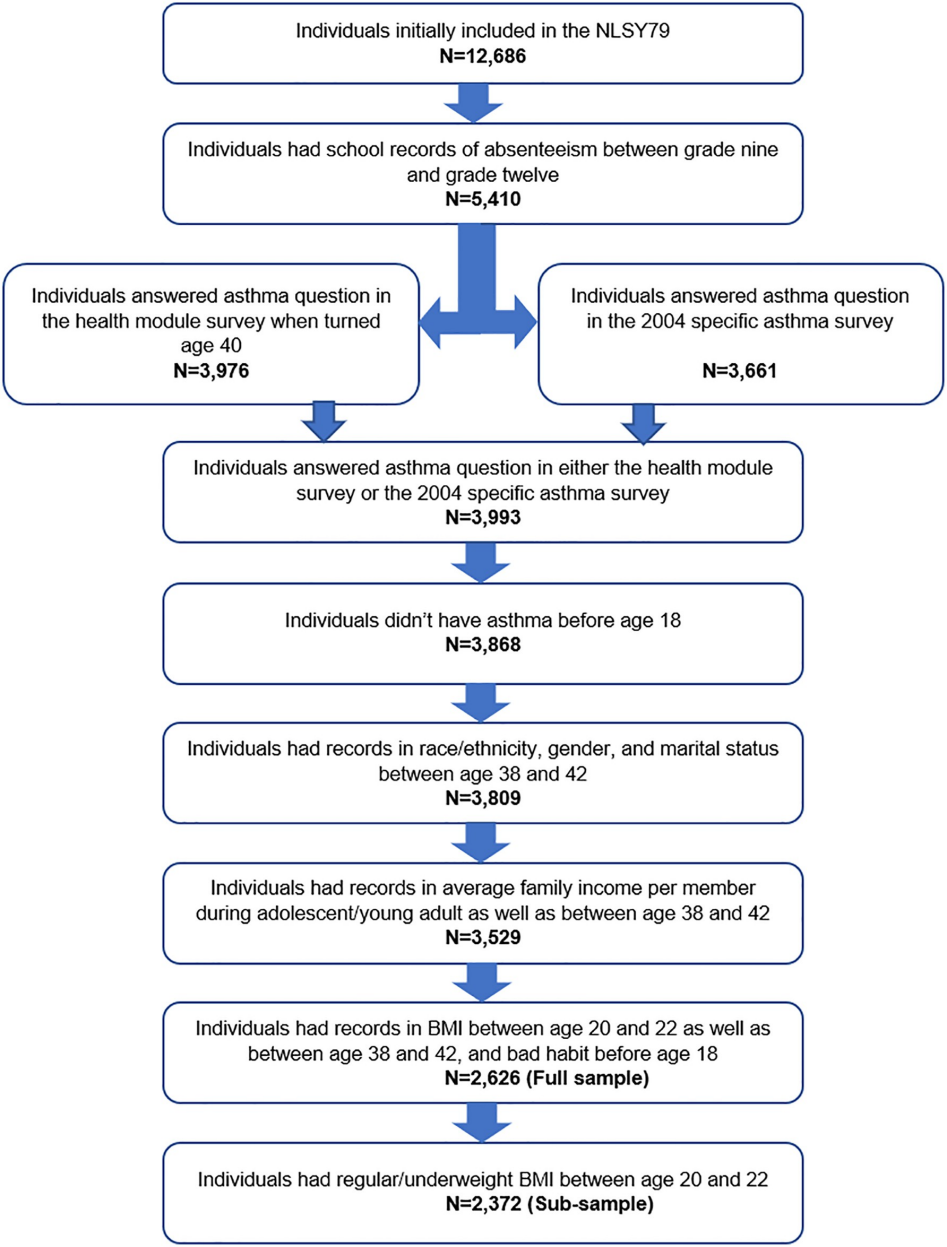
Sample selection process.

Fig 2 showed the overall sample selection process of the study. Beginning in 1979, the survey initially had 12,686 participants. Among them, 5,410 individuals had school records of absenteeism between grade nine and grade twelve. This study then included 3,993 cohort members who answered the asthma question either in the health module survey when they turned age 40 or another specific asthma survey in 2004. After excluding 125 individuals who had asthma before age 18, the sample size resulted in 3,868. The exclusion can maximize the possibility of intentional school absenteeism and control for the possibility that childhood asthma would cause school absenteeism. Thereafter, we included only those individuals who had complete records for the remaining variables of interest. The final full sample consisted of 2,626 cohort members. This study also created a sub-sample by excluding 254 individuals with overweight/obese BMI when they were 20–22 years old to control for potentially high initial health risks. The sub-sample consisted of 2,372 cohort members.

### Variables of interest

The outcome variable is whether a cohort member had adult-onset asthma at age 40 or in the mid-40s, as represented by a dichotomous (yes or no) variable. This variable was derived using either (i) the asthma item in the 40-and-over health module survey when the participants turned to age 40 or (ii) the asthma items in the specific asthma survey conducted in 2004 when nearly all participants were at age 40 or in the mid-40s. This variable was measured using either (i) the 40-and-over health module survey question indicating whether the cohort member had asthma; or (ii) the 2004 asthma specific survey indicating whether the cohort member currently has asthma, or the member was older than 40 during the last symptom of asthma. To study the intentional school absenteeism and to control for the possibility that childhood asthma would cause school absenteeism at young age; this study excluded cohort members who had asthma before age 18. The exclusion was based on the related asthma question asking the age first told having asthma in the 2004 specific asthma survey.

Our key explanatory variable is the number of years having CA for a cohort member, as represented by a count variable (minimum value = 0, maximum value = 4). Number of days absent in each year between grade nine and grade twelve were from the transcript survey reported by schools. Given that our cohort were enrolled in high school in the late 1970s and early 1980s, we used the pre-2016 definition of CA defined by the U.S. Department of Education: 15 or more days absent from school in an academic year. Hence, a cohort member who missed more than 15 days in a year was considered as having CA in that year. We assigned a 1/0 indicator [26] to each grade based on whether the cohort member had CA in that grade, then accumulated over the 4 years from grades nine to twelve as the measurement of number of years having CA for the cohort member (For example, an individual John could have CA in grades nine and eleven, so John’s cumulative years of having CA is two. Another individual Mary could have CA in grades ten and twelve, so Mary’s cumulative years of having CA is also two).

Our estimations included variables that were categorized as demographic (D), economic (E), and health (H) related. Explanatory variables in the demographic category were race/ethnicity, gender, marital status at age between 38 to 42, and the highest grade completed in middle age. Race/ethnicity included Black, Non-black non-Hispanic, and Hispanic. Marital status from the related survey question originally had three classifications. It was collapsed to two classifications in this study: never married/other situations, and married spouse present. Marital status reported at age between 38 to 42 was included. Highest grade completed was reported in the education related survey questions. Highest grade completed reported at age 42 was used to measure the highest grade completed in the middle age. It was converted to comparable aggregate measurement of educational attainment. Cohort members who completed less than 12^th^ grades were classified as “Less than 12^th^ Grade”. Individuals who reported finishing 12^th^ grade were grouped to “Twelfth Grade”. Those who completed less than 4^th^ year college were classified to “Less than Four-Year College”. Finally, individuals who completed at least 4^th^ year college were grouped to “At Least Four-Year College”.

Variables in the economic category were average family income per family member (AFIPFM) as an adolescent/young adult, and AFIPFM in mid-adulthood. AFIPFM in a given survey year was calculated using total net family income in a year divided by family size in the same year. Total net family income in each year was adjusted using consumer price index in all urban consumers (1983 dollar = 100). We first calculated the mean value of AFIPFMs when the individual’s age was between 14 and 26 and the mean value of AFIPFMs when the individual’s age was between 38 to 42. Then converted these two variables to categorical variables respectively using the following percentile classification: <33.3rd percentile, between 33.3rd percentile and 66.7th percentile, >66.7th percentile in each variable.

Explanatory variables in the health category were BMI classification in the age of early 20s, BMI classification in mid-adulthood, bad habit at age less than 18 years old, and whether one of the parents had asthma. We first derived BMI value in each survey year for everyone based on the survey questions about weight and height. Outlier/missing values were replaced by values imputed from adjacent years. BMI in the age of early 20s was calculated using mean value of BMIs when the individual was at age 20, 21 and 22. Before 1998, BMI index <27.8 for male and BMI index <27.3 for female were classified as “Normal/Underweight”; otherwise, classified as “Overweight”. Hence, we applied these old criteria on BMI in the age of early 20s. This variable was also used to exclude overweight/obese BMI individuals at their young age to create the sub-sample. BMI in mid-adulthood was measured as the mean of BMIs when the individual was at age between 38 to 42. The new criteria: BMI value <25 was used to classify “Normal/Underweight”; otherwise, classified as “Overweight”. Bad habit at age less than 18 years old was measured by either smoking daily or drinking regularly (2–3 times) per week in the related behaviour survey questions when the individual was less than age 18. Parental asthma was identified by the asthma health code (CDC–National Vital Statistics System 113 List). We created a binary indicator variable which assigned 0 or 1 if asthma appeared as one of the reported major health problems from either biological parent.

### Statistical methods

The logistic models can be represented by the logit function:

$$\text{Pr}\left(Y=1|\text{Absence},D,\;E,\;H\right)=\frac{exp(\beta_{0}+\beta_{1}\text{Absence}+B_{2}D+B_{3}E+B_{4}H\text{)}}{1+exp(\beta_{0}+\beta_{1}\text{Absence}+B_{2}D+B_{3}E+B_{4}H\text{)}},$$

where *Y* indicates whether an individual had adult-onset asthma in middle age, *Absence* is the number of years chronically absent between grades nine and twelve. *D*, *E*, *H* are variables in the demographic, economic, and health categories. For detailed descriptions of regression models, please see S1 Appendix in S1 File.

To calculate the expected costs of adult-onset asthma in middle age associated with high school absenteeism, we generated the predicted probability of having adult-onset asthma in middle age at a different number of years of CA from the logistic regression, holding all other variables constant at their mean values. We also estimated marginal effect with one additional year of CA on the probability of having adult-onset asthma in middle age.

Because there were a relatively large number of covariates, we examined multicollinearity among all independent variables using the variance inflation factor (VIF). In addition, we computed the tetrachoric/polychoric correlation between number of years having CA and the classifications of highest school grade completed in middle age. We compared descriptive statistics using Chi-square test. Statistical significance was defined as P ≤0.100.

### Methods of calculating costs of adult-onset asthma in middle age due to intentional high school absenteeism

Given that our cohort members turned age 40 years old around 2002 when adult-onset asthma was reported, we quantified the costs of asthma in 2002 for these individuals who were in high school during the late 1970s and early 1980s (consider the mid-point, year 1980). We considered all costs of asthma: direct medical costs (such as healthcare utilization and medications) and indirect costs (such as time lost from work and school) estimated by the study [24].

The costs of adult-onset asthma in middle age due to intentional high school absenteeism in 1980 was calculated as the following (S1 Appendix in S1 File provided detailed description of the estimation process). First, we estimated average costs of asthma per person in age 35–44 individuals with asthma. Then, we calculated the number of high school students who did not report childhood asthma before age 18. Finally, we calculated incremental/marginal costs using the following formula:

$${\text{IC}}_{ij}=\left(p_{i}-p_{j}\right)\times \text{hs(non-childhood-asthma)}\times AV\text{C},$$

where *IC*_*ij*_ is incremental costs of going from CA level *j* to *i*. *p*_*i*_ is the predicted probability of adult-onset asthma in middle age at level *i* of CA, (*p*_*i*_ − *p*_*j*_) is the marginal/incremental probability going from CA level *j* to *i*. hs(non-childhood-asthma) is the number of high school students who did not report childhood asthma before age 18. AVC is the average costs of asthma per person in age 35–44 individuals with asthma in 2002.

Accumulating these marginal costs gives the costs of asthma incurred by different levels of CA. We measured the magnitude of asthma costs due to intentional high school absenteeism by calculating the share of the asthma costs in selected countries’ gross domestic products (GDPs) and costs of illness. We select a couple of countries’ GDPs in 2002, including the U.S., Brazil, Colombia, Ghana, Chad as well as the total costs (both direct and indirect costs) of illness for Canada in 1998. All monetary units are quoted in 2002 U.S. dollars.

### Sensitivity analyses—Methods

Additionally, we conducted two sensitivity analyses to assess the robustness of our findings. Frist, the descriptive statistics of our samples showed that at least 93% of individuals not having adult-onset asthma in middle age, which indicated our outcome variable had a high proportion of zeros. Therefore, we ran zero-inflated binomial regression models on the data. Secondly, we changed our definition of CA from the Department of Education’s pre-2016 definition to the post-2016 definition. The pre-2016 used the threshold of 15 days. In 2016, the Department of Education changed the definition of CA to 10% of school days absent in a year. The number of high school instructional days in the late 1970s and early 1980s was documented to range from 178 to 179 days [27], which led to a threshold of 18 days for CA. This new threshold is more stringent compared to the pre-2016 definition. Therefore, we used this 18-days new threshold in the sensitivity analyses. Hence, an individual who missed more than 18 days in a year was considered as having CA in that year. We assigned a 1 or 0 indicator to each grade based on whether the cohort member had CA (new threshold) in that grade, then accumulated over the 4 years from grades nine to twelve as the measurement of number of years having CA (new threshold) for the cohort member.

All analyses were performed between December 1, 2023 and January 31, 2024 using SAS software, version 9.4 (SAS Institute, Inc. Cary, NC), and STATA 15 (StataCorp. 2017. Stata Statistical Software: Release 15. College Station, TX: StataCorp LLC.).

## Results

### Descriptive analysis

Recall, we started with a total of 12,686 NLSY79 participants. Our data filtering process resulted in 2,626 for the full sample and 2,372 when we eliminate individuals with overweight BMI at young age for the sub-sample.

Table 1 provides descriptive statistics for our full sample (N = 2,626) and sub-sample (N = 2,372). The descriptive statistics in two samples were essentially the same except for BMI in mid-adulthood. Approximately 6.5% in both samples had adult-onset asthma at age 40 or in the mid-40s. This was slightly lower than the national average of 6.8% among adult 18 years and older, and 7.2% among total U.S. population in 2002 reported by the CDC’s Asthma Prevalence, Health Care Use and Mortality: United States, 2002 (https://www.cdc.gov/nchs/data/hestat/asthma/asthma.htm).

**Table 1:** Descriptive statistics of the cohort members in the full sample and sub-sample of the study.

Characteristics	Full Sample		Sub-Sample^a^		P-Value^b^
	(N = 2626)		(N = 2372)		
	n	%	n	%	
Had Asthma at Age 40 or in the Mid-40s					
Yes	176	6.7	155	6.5	0.812
No	2450	93.3	2217	93.5	
Number of Years Having Chronic Absenteeism					
0	1279	48.7	1175	49.5	0.971
1	636	24.2	570	24.0	
2	411	15.7	368	15.5	
3	196	7.5	171	7.2	
4	104	4.0	88	3.7	
Chronic Absenteeism in Grade Nine					
Yes	569	21.7	489	20.6	0.363
No	2057	78.3	1883	79.4	
Chronic Absenteeism in Grade Ten					
Yes	657	25.0	581	24.5	0.668
No	1969	75.0	1791	75.5	
Chronic Absenteeism in Grade Eleven					
Yes	633	24.1	563	23.7	0.760
No	1993	75.9	1809	76.3	
Chronic Absenteeism in Grade Twelve					
Yes	603	23.0	538	22.7	0.813
No	2023	77.0	1834	77.3	
**Demographic Category (D)**					
Race/Ethnicity					
Black	769	29.3	683	28.8	0.894
Non-Black, Non-Hispanic	1567	59.7	1431	60.3	
Hispanic	290	11.0	258	10.9	
Gender					
Male	1315	50.1	1193	50.3	0.877
Female	1311	49.9	1179	49.7	
Marital Status in Mid-adulthood					
Never Married/Other	1023	39.0	902	38.0	0.500
Married, Spouse Present	1603	61.0	1470	62.0	
Highest Grade Completed in Middle age					
Less than Twelfth Grade	167	6.4	148	6.2	0.833
Twelfth Grade	1179	44.9	1040	43.8	
Less than Four-Year College	625	23.8	568	23.9	
At Least Four-Year College	655	24.9	616	26.0	
**Economic Category (E)**					
Average Family Income Per Member During Adolescent/Young Adult					
< 33.3rd Percentile	882	33.6	789	33.3	0.729
Between 33.3rd and 66.7th Percentile	858	32.7	758	32.0	
> 66.7th Percentile	886	33.7	825	34.8	
Average Family Income Per Member in Mid-adulthood					
< 33.3rd Percentile	874	33.3	789	33.3	1.000
Between 33.3rd and 66.7th Percentile	876	33.4	791	33.3	
> 66.7th Percentile	876	33.4	792	33.4	
**Health Category (H)**					
BMI When Age in the 20, 21 and 22					
Normal/Underweight	2372	90.3	2372	100.0	-
Overweight	254	9.7	-	-	
BMI in Mid-adulthood					
Normal/Underweight	875	33.3	874	36.8	<0.010
Overweight	1751	66.7	1498	63.2	
Bad Habit When Age<18					
No	1194	45.5	1068	45.0	0.753
Yes	1432	54.5	1304	55.0	
Either One of Parents had Asthma					
Yes	42	1.6	40	1.7	0.809
No	2584	98.4	2332	98.3	

^a^ Our sub-sample excluded cohort members with overweight and obese BMI when they were young to control for potentially high initial health risks.

^b^ Calculated by Chi-square test.

Table 1 also showed that about 50% had a least one year of CA between grades nine and twelve (about 21%-25% had CA within each grade). About 40% were Black or Hispanic. Both samples were evenly split by gender. When the individuals were in their mid-adulthood in both samples, about 60% were married with spouse present, 45% completed twelfth grade while 25% completed at least four years of college. When the individuals were between 20 and 22 years old, approximately 10% were considered overweight. BMI classification in mid-adulthood showed that the full sample had a larger proportion of overweight than that of sub-sample (66% vs. 63%, P<0.010). Approximately 45% were smoking daily or drinking regularly every week before age 18. Lastly, about 1.5% of the individuals reported that at least one of their biological parents had asthma as a chronic health problem.

### Logistic regression estimations

Table 2 shows that using the full sample, more years of CA in high school were associated with a significant (P = 0.100) increased risk of adult-onset asthma in middle age (OR = 1.12; 95% CI, 0.98–1.28). Males had a lower risk of adult-onset asthma than females (OR = 0.31; 95% CI, 0.22–0.45; P<0.010) in mid-adulthood. Individuals who never married or in other situations in their mid-adulthood had a higher risk of adult-onset asthma than those who were married with spouse present in middle age (OR = 1.40; 95% CI, 1.00–1.96; P = 0.054). There were no significant impacts by education and economic variables. Among health variables, individuals with normal/underweight BMI in mid-adulthood had a significantly (P <0.010) lower risk of adult-onset asthma than their overweight counterparts in mid-adulthood (OR = 0.52; 95% CI, 0.36–0.76). Participants with no bad habits before age 18 had a lower risk of asthma in adulthood (OR = 0.73; 95% CI, 0.53–1.01; P = 0.057). If either one of biological parents had asthma, the individuals would have a significantly (P<0.100) higher risk of adult-onset asthma in middle age than those who did not report their parents having asthma (OR = 2.17; 95% CI, 0.88–5.38).

**Table 2:** Logistic regression model with adult-onset asthma in middle age as the dependent variable and number of years of chronic absenteeism as the key explanatory variable.

Variable	Full Sample			Sub-Sample^c^		
	OR^a^	95% CI^b^	P-value	OR^a^	95% CI^b^	P-value
**Key Explanatory Variable**						
Number of Years Having Chronic Absenteeism	1.12	(0.98, 1.28)	0.103	1.15	(1.00, 1.33)	0.057
**Demographic Category (D)**						
Race/Ethnicity						
Black	0.82	(0.49, 1.38)	0.457	0.72	(0.42, 1.24)	0.231
Non-Black, Non-Hispanic	0.67	(0.41, 1.09)	0.107	0.64	(0.38, 1.08)	0.097
Hispanic	1			1		
Gender						
Male	0.31	(0.22, 0.45)	<0.010	0.32	(0.22, 0.46)	<0.010
Female	1			1		
Marital Status in Mid-adulthood						
Never Married/Other	1.40	(1.00, 1.96)	0.054	1.60	(1.12, 2.30)	0.010
Married, Spouse Present	1			1		
Highest Grade Completed in Middle Age						
Less than Twelfth Grade	1.19	(0.60, 2.36)	0.613	0.85	(0.38, 1.93)	0.700
Twelfth Grade	0.86	(0.56, 1.32)	0.486	0.97	(0.62, 1.53)	0.906
Less than Four-Year College	0.69	(0.43, 1.11)	0.125	0.75	(0.45, 1.24)	0.265
At Least Four-Year College	1			1		
**Economic Category (E)**						
Average Family Income Per Member During Adolescent/Young Adult						
< 33.3rd Percentile	0.75	(0.48, 1.17)	0.207	0.85	(0.53, 1.37)	0.510
Between 33.3rd and 66.7th Percentile	0.93	(0.63, 1.38)	0.716	0.96	(0.63, 1.46)	0.847
> 66.7th Percentile	1			1		
Average Family Income Per Member in Mid-adulthood						
< 33.3rd Percentile	0.92	(0.60, 1.41)	0.692	0.72	(0.46, 1.13)	0.152
Between 33.3rd and 66.7th Percentile	0.93	(0.62, 1.40)	0.734	0.85	(0.56, 1.31)	0.462
> 66.7th Percentile	1			1		
**Health Category (H)**						
BMI When Age in the 20, 21 and 22						
Normal/Underweight	1.01	(0.61, 1.67)	0.962	-	-	-
Overweight	1			-	-	-
BMI in Mid-adulthood						
Normal/Underweight	0.52	(0.36, 0.76)	<0.010	0.50	(0.34, 0.73)	<0.010
Overweight	1			1		
Bad Habit When Age<18						
No	0.73	(0.53, 1.01)	0.057	0.68	(0.48, 0.96)	0.029
Yes	1			1		
Either One of Parents had Asthma						
Yes	2.17	(0.88, 5.38)	0.094	1.94	(0.72, 5.18)	0.189
No	1			1		

^a^ OR: Odd Ratios.

^b^ CI: Confidence Interval.

^c^ Our sub-sample excluded cohort members with overweight and obese BMI when they were young to control for potentially high initial health risks.

All notable associations in the full sample were confirmed in the sub-sample, with some reporting stronger association. As shown in Table 2, the association between years of CA in high school and adult-onset asthma in middle age was marginally significant at P = 0.050 level. The results also show that non-Black and non-Hispanic individuals had a lower risk of adult-onset asthma than Hispanics in middle age (OR = 0.64; 95% CI, 0.38–1.08), which was significant at the P = 0.100 level (P = 0.097). Individuals’ marital status in their mid-adulthood was significant at the P = 0.010 level. Participants’ bad habits before age 18 was significant at the P = 0.050 level (P = 0.029). One exception is that one of parents had asthma is no longer significant in the sub-sample estimations. This implies that individuals who reported their parents having asthma were mostly those classified as overweight/obese at age between 20 and 22, who were excluded in the sub-sample.

The multicollinearity test among all independent variables using the variance inflation factors (VIFs) generated all VIF values below 1.50, which indicated a lack of multicollinearity in the logistic regressions. Additionally, the tetrachoric/polychoric correlation between number of years having CA and the classifications of highest school grade completed in mid-adulthood was about -0.30 in both samples, which indicated a negative relationship: more years of CA were related to lower education levels.

### Predicted probabilities of adult-onset asthma in middle age and marginal effects

Predicted probabilities of adult-onset asthma in middle age based on the logistic regression model are shown in Table 3. When varying the number of years having CA from 0 to 4, we showed the predicted probability under different hypothetical scenarios. For instance, when the number of years having CA is 0, that is, the hypothetical scenario where all individuals in the cohort did not experience any high school absenteeism. When the number of years having CA is 1, that is, another hypothetical scenario where all individuals in the cohort experienced one year of high school absenteeism. In Table 3, the full sample predictions show that individuals who did not have CA from grade nine to grade twelve were the least likely to have adult-onset asthma in middle age (0.050). The predicted probability increased to 0.056, 0.062, 0.069 and 0.077 for individuals who had one, two, three and four years of CA, respectively (P<0.010 for all levels of CA). The average marginal effect indicated an important dose-dependent relationship: with one additional year of CA between grade nine and twelve, the probability of having adult-onset asthma in middle age increased by 0.006 (P = 0.100).

**Table 3:** Predicted probability of adult-onset asthma in adulthood at specific number of years of chronic absenteeism and average marginal effect.

Number of Years Having Chronic Absenteeism	Full Sample		Sub-Sample^a^	
	Predicted Probability	95% CI^b^	Predicted Probability	95% CI^b^
0	0.050	(0.039, 0.061)^c^	0.047	(0.036, 0.058)^c^
1	0.056	(0.046, 0.065)^c^	0.054	(0.044, 0.064)^c^
2	0.062	(0.049, 0.075)^c^	0.062	(0.048, 0.075)^c^
3	0.069	(0.048, 0.089)^c^	0.070	(0.048, 0.092)^c^
4	0.077	(0.046, 0.108)^c^	0.080	(0.046, 0.114)^c^
Average Marginal Effect	0.006	(-0.001, 0.013)^d^	0.007	(0.000, 0.014)^e^

^a^ Our sub-sample excluded cohort members with overweight and obese BMI when they were young to control for potentially high initial health risks.

^b^ CI: Confidence Interval.

^c^ P-value<0.010.

^d^ P-value = 0.102.

^e^ P-value = 0.056.

Similar results were present in the sub-sample. Individuals who did not have any CA from grade nine to grade twelve were the least likely to have adult-onset asthma in middle age (0.047). The predicted probability increased to 0.054, 0.062, 0.070 and 0.080 for individuals who had one, two, three and four years of CA, respectively (P<0.010 for all levels of CA). Particularly, dose-dependent relationship became 0.007 (P = 0.056).

The model-based predicted values are consistent with the descriptive statistics (Table 1): about 6.5% of cohort members had adult-onset asthma at age 40 or in the mid-40s in both full sample and sub-sample. That is the unconditional probability 6.5% of having adult-onset asthma in middle age. From our empirical model, if all individuals had two or less years of CA, the probability of having adult-onset asthma in middle age would be lower than the unconditional probability. Only three or four years of CA generated probabilities greater than the unconditional probability. The model-based predicted values are also consistent with the point estimate for the key explanatory variable in Table 2, that is, the likelihood of having adult-onset asthma in middle age increased with additional years of CA.

### Quantifying costs of adult-onset asthma in middle age due to intentional high school absenteeism

The costs of adult-onset asthma in middle age attributed to intentional high school absenteeism are presented in Table 4.

**Table 4:** Quantify costs of adult-onset asthma in middle ages at specific number of years of chronic absenteeism.

Number of Years Having Chronic Absenteeism in High School	Predicted Probability of Adult-onset Asthma in Middle Age	Marginal/ Incremental Probability	Marginal/ Incremental Asthma Costs (2002 US Dollars)	Costs above Baseline (2002 US Dollars)
(E)	(F)	(G)	(H)^a^	(I)^b^
**Full Sample**				
0	0.050	Baseline	Baseline	Baseline
1	0.056	0.006	$175,084,282	$175,084,282
2	0.062	0.006	$193,519,099	$368,603,381
3	0.069	0.007	$213,596,008	$582,199,389
4	0.077	0.008	$235,376,976	$817,576,365
**Sub-Sample** ^c^				
0	0.047	Baseline	Baseline	Baseline
1	0.054	0.007	$208,174,003	$208,174,003
2	0.062	0.008	$235,779,754	$443,953,757
3	0.070	0.009	$266,514,775	$710,468,532
4	0.080	0.010	$300,533,983	$1,011,002,515

^a^ Column (H) = High school students in 1980 (13,189,819) × Column (G). × Average asthma costs in age group 35–44 in 2002 ($2,349).

^b^ Column (I) is the cumulative costs of Column (H).

^c^ Our sub-sample excluded cohort members with overweight and obese BMI when they were young to control for potentially high initial health risks.

We first estimated the average costs of asthma per person in age 35–44 individuals with asthma, $2,349 in 2002 U.S dollars. The detailed process was described in S1 Appendix in S1 File. The total student enrollment between grade nine and twelve in 1980 was 13,616,000, which was reported in the historical summary of public elementary and secondary school statistics from National Center for Education Statistics (https://nces.ed.gov/programs/digest/d08/tables/dt08\_032.asp). In our sample selection process, among 3,993 included cohort members who answered the asthma survey questions, we excluded 125 individuals who had asthma before age 18 in order to maximize the intentional (non-childhood-health related) school absences and control for the possibility that teenagers’ asthma would cause school absenteeism. This proportion was calculated as 3.13% (125/3993). Therefore, we applied this proportion to calculate the number of high school students in 1980: (1–3.13%) × 13,616,000 = 13,189,819. These were individuals who were in high school in 1980, and their health in mid-adulthood could be possibly impacted by intentional high school absenteeism.

Using the predicted probability of adult-onset asthma in middle age in Table 3, we calculated the marginal/incremental probability of going from one level of CA to the next, which was shown in column (G) of Table 4. Using the marginal probability, along with the 13,189,819 high school students in 1980, and $2,349 average asthma costs in age group 35–44 in 2022, we calculated the marginal/incremental costs of asthma as shown in column (H). Accumulating the marginal costs of asthma gives costs above baseline for each level of CA, as shown in column (I). For example, using the full sample, if all high school students had 4 years of CA in the early 1980s it would result in total costs of $817,576,365 in 2002. Similar calculations using the sub-sample resulted in a 2002 expected costs of $1,011,002,515 for the topmost CA.

These expected asthma costs were also shown in column (I) of Table 5. We selected a couple of countries, including the U.S. (GDP: $10.94 trillion), Brazil (GDP: $508 billion), Colombia (GDP: $97.96 billion), Ghana (GDP: $6.166 billion), Chad (GDP: $1.997 billion). These countries’ GDP values were measured using the 2002 U.S. dollars (USD). In addition, the total costs (both direct and indirect costs) of illness for Canada in 1998 were measured as $174.7 billion 2002 Canadian dollars (CAD), which was equivalent to $111.349 billion 2002 USD using the average exchange rate (1 CAD = 0.637 USD) conversion. The selected countries’ GDPs and costs of illness were included in column (K) of Table 5. The expected costs of adult-onset asthma in middle age due to intentional high school absenteeism were 0.007%/0.009% of the US GDP in 2002, and 0.161%/0.199%, 0.835%/1.032%, 13.259%/16.396%, 40.940%/50.626% of the 2002 GDP of Brazil, Colombia, Ghana, Chad, respectively. Additionally, the expected costs of adult-onset asthma in the middle age due to high school absenteeism were 0.734%/0.908% of the total costs of illness for Canada in 1998. These calculated shares/fractions were shown in column (L) of Table 5.

**Table 5:** Magnitude of costs of adult-onset asthma in middle age due to high school chronic absenteeism.

Number of Years Having Chronic Absenteeism in High School	Costs above Baseline	Selected Country’s GDP^a^/costs of illness	Values in 2002 US Dollars	Share/ Fraction
(E)	(I)	(J)	(K)	(L)^b^
**Full Sample**				
4	$817,576,365	USA’s GDP in 2002	$10,940,000,000,000	0.007%
		Brazil’s GDP in 2002	$508,000,000,000	0.161%
		Colombia’s GDP in 2002	$97,960,000,000	0.835%
		Ghana’s GDP in 2002	$6,166,000,000	13.259%
		Chad’s GDP in 2002	$1,997,000,000	40.940%
		Canada’s total direct and indirect costs of illness in 1998	$111,349,304,699	0.734%
**Sub-Sample** ^c^				
4	$1,011,002,515	USA’s GDP in 2002	$10,940,000,000,000	0.009%
		Brazil’s GDP in 2002	$508,000,000,000	0.199%
		Colombia’s GDP in 2002	$97,960,000,000	1.032%
		Ghana’s GDP in 2002	$6,166,000,000	16.396%
		Chad’s GDP in 2002	$1,997,000,000	50.626%
		Canada’s total direct and indirect costs of illness in 1998	$111,349,304,699	0.908%

^a^ GDP: Gross Domestic Product.

^b^ Column (L) = Column (I) / column (K).

^c^ Our sub-sample excluded cohort members with overweight and obese BMI when they were young to control for potentially high initial health risks.

### Sensitivity analyses

In the first sensitivity analysis, we ran zero inflated binomial regression models, and we found very similar relationship between high school CA and adult-onset asthma in middle age. The estimate results were shown in S1 Appendix in S1 File. In the second sensitivity analysis, we changed the threshold for defining CA from benchmark’s threshold of 15 days to a new threshold of 18 days. Nearly 40% of the cohort members had a least one year of CA (new threshold) between grades nine and twelve. This percentage is smaller than that in the results using the benchmark threshold of 15 days. Hence, the new threshold of 18 days is more stringent. The regression results were very similar to those in estimations using the threshold of 15 days. The estimate results were shown in S1 Appendix in S1 File.

## Discussion

The long term detrimental consequences of CA on health in mid-adulthood are well documented [14, 25]. CA frequently impedes scholastic performance, leading to a decrease in educational attainment or being disconnected from school, and it also tends to anticipate harmful health conditions and behaviors in later life. Additionally, a lesser level of education is typically linked to future unemployment and poorer income, both of which are linked to more serious health issues. However, there is a dearth of empirical studies evaluating the direct association between school absence and mid-adulthood health condition. We investigated three possible associations in this study. First, is CA during high school directly related to adult-onset asthma in middle age? Second, is health-risk activities during adolescent (drinking and smoking) linked to adult-onset asthma in middle age? Third, are income levels in early or middle life correlated with adult-onset asthma in middle age? By examining our hypotheses with a sizable sample from the NLSY79 national longitudinal data, we aimed to close the gap in the literature. Additionally, we devised a technique to quantify the economic burden of adult-onset asthma in middle age under various scenarios of deliberate CA in high school. Whether a cohort member had adult-onset asthma at age 40 or in their mid-40s was the study’s outcome variable. The number of years having CA from grade nine to grade ten was the primary explanatory variable. The control variables, which comprised demographic, economic, and health characteristics, were applied to our regression models. Our findings supported the hypothesis that deliberate high school absences have a direct detrimental effect on adult-onset asthma in middle age. Regression models were used to predict the likelihood of adult-onset asthma in middle age when no one had CA in high school and the likelihood of adult-onset asthma in middle age when everyone had one, two, three, or four years of CA, respectively. Ultimately, we were able to calculate the total costs of adult-onset asthma in middle age owning to the different levels of CA.

According to the outcome variable’s descriptive statistics, roughly 6.5% of the participants in our cohort who were included had adult-onset asthma at or around the age of 40. This percentage was extremely similar to the 6.8% national average for adults 18 years of age and older in the United States as reported by the CDC’s Asthma Prevalence. The following are the main conclusions from our investigation. First, our model estimates demonstrated a direct correlation between the number of years of having CA during high school and the chance of having adult-onset asthma in middle age. Crucially, this conclusion persisted even after taking unhealthy behaviors and lower family income into consideration. Consequently, our results showed a strong negative correlation between regular absence in high school and long-term mid-adulthood health outcomes, a finding that calls for further study and policy consideration. Second, the chance of having adult-onset asthma in middle age would be reduced for those who did not engage in risky habits throughout their adolescence, such as smoking cigarettes every day or drinking alcohol frequently (two to three times per week). People who developed regular drinking or smoking habits as children were prone to continue these high-risky activities into old life. The current research has indicated that adult-onset asthma is caused by smoking. Drinking alcohol has a mixed impact on asthma. According to one study [28], drinking alcohol appeared to be a significant asthmatic response trigger. According to a different study [29], adults who drink infrequently had the highest risk of having adult-onset asthma, while those who drink alcohol once a week had the lowest risk. Our findings may be explained by the possibility that CA causes school dropout or disengagement, which would link to unhealthy behaviors in those affected and ultimately result in adult-onset asthma in mid-adulthood. A person with less education or who is not involved in school is more likely to miss out on opportunities to learn from peers and teachers of how to form healthy habits and become health literate. Regular attendance at school has been demonstrated to be beneficial for one’s health trajectory. Third, there was no indication that one’s income level was related to having adult-onset asthma in middle age. We demonstrated that the highest school grade attained in mid-adulthood and the number of years with CA had a tetrachoric/polychoric correlation of roughly -0.30, indicating a negative relationship between CA and educational attainment. Consequently, there was a higher likelihood of high school dropout for those with CA. Reduced income levels can result from lower income levels due to higher unemployment rates, lower status occupations, and lower educational attainment. However, our findings did not support a link between income level and adult-onset asthma in middle age.

The aforementioned correlations were examined while adjusting for demographic and additional risk factors. Consequently, our investigation also yields the following conclusions. First, compared to Hispanic people, non-Black non-Hispanic people were less likely to have adult-onset asthma in middle age. According to the CDC’s asthma prevalence, the prevalence of asthma among adult Hispanics in the United States has generally been lowest in recent years. Our results differed, most likely as a result of the late 1970s selection of cohort members. Second, compared to females, men were less likely to have adult-onset asthma in middle age. Our results were in line with the literature on adult-onset asthma. For example, a U.S. longitudinal study [30] discovered that by the age of 40, women had a higher percentage of adult-onset asthma than men did. Third, compared to those who were married with a spouse present in middle age, those who were single or in non-matrimonial situations had an increased risk of adult-onset asthma. According to a Finnish longitudinal study [31], stressful life events may hasten the onset of asthma. They discovered that marital issues and separation or divorce were two of the top ten stressful life events linked to the onset of asthma. Fourth, compared to those who were overweight, those with a normal or underweight BMI in mid-adulthood had a lower chance of having adult-onset asthma in middle age. Our findings supported a substantial body of research showing obesity to be a significant risk factor for adult-onset asthma. According to a multi-year study [32], obese individuals in the age groups of 20–39, 40–59, and 60 and over were all linked to a higher risk of asthma. Finally, compared to those who did not report their parents’ having asthma, those who had asthma in either of their biological parents would be at a higher risk of having adult-onset asthma in their middle age. Our results were consistent with the literature. For example, in one study [33], a family history of asthma is a significant risk factor for asthma.

In addition, we developed a method to quantify total costs of adult-onset asthma in middle age due to no high school CA and different levels of high school CA. The costs of adult-onset asthma due to the topmost high school absenteeism could result in approximately $1 billion in 2002. These costs are relatively large considering high school students constituted only 6% of the U.S. population (In 1980, the U.S. population was 226,545,805 according to the U.S. Census Bureau. The total student enrollment between grade nine and twelve in 1980 was 13,616,000 in the same year). Our findings highlighted important barriers in the pathway and etiology of asthma. These served as significant intervention points. Even without altering the current curricula, public policy that promotes regular attendance at high school could reduce the economic burden of adult-onset asthma in the future. This is comparable to the idea that policies affecting educational attainment could significantly affect population health [19].

We used the same data to run a zero-inflated binomial model as a robustness check, and the results were remarkably similar. Furthermore, there was little difference in the estimation results when the benchmark’s 15-day threshold for defining CA was replaced with a new 18-day threshold. The model retained the significant direct correlation between high school CA and having adult-onset asthma in middle age.

The findings in our study are subject to several limitations. First, the association between high school CA and having adult-onset asthma in middle age may not be the actual causal relationship. The same limitation also applied to the association between health-risk behaviors in the adolescence and having adult-onset asthma in middle age, and the association between the number of years having high school CA and the classifications of the highest school grade completed in mid-adulthood. Additional analyses are needed to assess the true causal relationship. Nonetheless, our regression models simultaneously incorporated multiple hypotheses, controlled for known risk factors, ran on longitudinal data over a 20-year period. The estimated results consistently showed that more years of CA in high school were associated with an increased risk of adult-onset asthma in middle age. Second, rather than using a diagnosis from a medical record, our measurements to determine whether the participants or their parents had asthma were self-reported. The self-reported data was gathered from questionnaire answers about the health conditions of the respondents or their parents. However, asthma is characterized by inflammation, hyperresponsiveness, or airway blockages. Additionally, according to the Asthma and Allergy Foundation of America (https://aafa.org/asthma/asthma-diagnosis/), diagnosing asthma involves a personal and medical history, a physical examination, lung function tests, allergy tests, and blood tests. Because of this, it is difficult to misdiagnose asthma, and those who answered the questionnaires were almost certainly suffering from the condition. The third limitation is that we made the reasonable assumption that a pattern of frequent absences from school indicates non-connectedness to school. But it does imply that regular school attendance denotes a connection to school. This could be a strong assumption given that we abstract from the mental states of school attendees. Finally, we did not account for the quality of formal education, which may have a bearing on the occurrence of diseases in the future, and instead only measured the quantity of it. As a result, we might be underestimating the impact of low education on income or health-risk behaviors.

Notwithstanding these drawbacks, the study has a lot of advantages. First off, a sizable national longitudinal data set spanning 20 years served as the foundation for our investigation. As far as we are aware, this study is one of the few that looked into the relationship between high school absence and long-term health outcomes. Second, we simultaneously tested our newly proposed and preexisting hypotheses regarding the relationship between long-term health outcomes that are adversely affected by school absences and education attainments. Third, we used two different definitions of CA in our analyses, respectively. The outcomes were strikingly alike. Fourth, under various high school CA scenarios, our regression models produced predicted probabilities of adult-onset asthma in middle age. The estimated probability closely matched the adult prevalence of asthma in the United States. Lastly, we devised a way to quantify the economic costs associated with adult-onset asthma in middle age brought on by willful high school absences. Our study offered a way to quantify the economic burden that results from repeated absences from high school.

## Conclusion

The primary objective of our study was to investigate the potential association between CA in high school and adult-onset asthma. As a corollary, we also examined two potential channels through which CA in high school may influence adult-onset asthma in middle adulthood. First, CA can hinder learning, reduce educational success, and cause individuals to disengage with the educational system. For this reason, these people have higher risk factors, including obesity, smoking and alcohol consumption. All of these are linked to adult-onset asthma in middle age. Second, the risk of dropping out of school is higher for people with CA. This can lead to future unemployment and reduced income, exposing them to environmental allergens and causing adult-onset asthma in middle age. After adjusting for health-risk behaviors and education-income channels, our empirical results continue to show a strong inverse relationship between intentional CA in high school and asthma in middle-aged adults. Additionally, our findings suggest that abstaining from harmful habits such as smoking regularly or drinking alcohol frequently at a young age would reduce the risk of adult-onset asthma in middle adulthood. Lastly, income level does not play a role in the association.

Futhermore, we designed a method based on the expected probabilities from our empirical estimates to measure the potential costs of asthma in middle-aged adults and study the dosage relationship between expected probabilities and years of high school CA. According to our empirical analysis, asthma-related costs in the United States, ranging from $817 million to $1 billion in 2002, can potentially be attributed to individuals with four years of high school CA from the late 1970s to early 1980s. Given that only 6% of Americans attended high school in 1980, these costs were particularly significant.

Our empirical study fills a gap in the literature and merits further research and policy consideration. Our research shows that adolescents who regularly attend school have a reduced incidence of adult-onset asthma in middle age, thereby reducing the future economic burden of asthma on society. In addition, our study highlights possible limitations, underscoring the importance of future research examining the relationship between school absence and health problems in adulthood. For example, data from a more modern cohort would be useful to confirm whether our current results hold.

## Supporting information

S1 File (DOCX)
