# Supplementary material for: Adult-onset asthma morbidity and related economic costs in middle age due to intentional chronic absenteeism in high school: An epidemiologic study using the national longitudinal survey of youth 1979 data
Source: PLoS One. 2024 Aug 2;19(8):e0306451. doi: 10.1371/journal.pone.0306451 (PMC11296655; doi:10.1371/journal.pone.0306451)
Supplement: S1 File — (DOCX) [file pone.0306451.s001.docx]

**S1 Appendix**

1. **Process of Quantifying Costs of Adult-onset Asthma in Middle Age Due to Intentional High School Absenteeism**

The National Longitudinal Survey of Youth 1979 (NLSY79) started in 1979 and initially included youths aged 14 to 22. The youths who enrolled in high school at that time would be between age 35 and 44 in 2002. The outcome variable in this study was whether an included NLSY79 cohort member had adult-onset asthma at age 40 or in the mid-40s. Therefore, we would need to estimate costs of asthma for age 35 and 44 individuals with asthma in year 2002.

**Step 1: Estimate the number for age 35-44 individuals with asthma**

Based on the 2002 BRFSS asthma prevalence data (https://www.cdc.gov/asthma/brfss/02/default.htm), we estimated the proportion of age 35-44 in all individuals (children and adults) who had current asthma. This proportion was estimated as 16.79%. This means that in 2002, among all individuals with current asthma, 16.79% was between age 35 and 44.

According to the results in the study [1], in 2002, the total number of individuals with asthma was estimated to be 11.6 million in the U.S.

Therefore, using the estimated proportion and total number of individuals with asthma, we calculated the number for age 35 and 44 individuals with asthma, 1.95 million (16.79% × 11.6 million).

**Step 2:** **Estimate the total costs for age 35-44 individuals with asthma**

According to the results in the study [1], in 2002, the total costs (both direct and indirect costs) of asthma was estimated to be 52.97 billion 2009 U.S. dollars (USD), which were equivalent to 44.43 billion of 2002 USD if we made conversion using the U.S. consumer price index. There were three components in the total costs of asthma: (i) direct costs, which was estimated to be 48.64 billion 2009 USD or 40.79 billion 2002 USD; (ii) costs of work and school days lost, which was estimated to be 1.67 billion 2009 USD or 1.40 billion 2002 USD; (iii) productivity loss due to death, which was estimated to be 2.66 billion 2009 USD or 2.23 billion 2002 USD.

Based on the National Health Expenditures Data by Age and Sex provided by Centers for Medicare & Medicaid Services, Office of the Actuary, National Health Statistics Group (https://www.cms.gov/data-research/statistics-trends-and-reports/national-health-expenditure-data/age-and-sex), in 2002, age group 19-44 accounted for 24.3% of the total personal health care spending. We assumed that total spending was evenly distributed within age group 19-44. Therefore, we estimated that age group 35-44 accounted for 9.35% of total personal health care spending. Based on this estimation, we further assumed that age 35-44 individuals with asthma also accounted for 9.35% of direct costs of asthma. Therefore, we calculated the direct costs of asthma for age 35-44 individuals with asthma, 3.81 billion 2002 USD (9.35% × 40.79 billion).

To estimate costs of work and school days lost, we assumed that these costs were evenly distributed among all ages. Hence, we calculated the costs of work and school days lost for 35-44 individuals with asthma, 0.24 billion in 2002 USD (16.79% × 1.4 billion).

To estimate productivity loss due to death in age group 35-44, we used the method in the study [1]. In 2002, there were 472 deaths with asthma as underlying causes for age 35-44 individuals with asthma, and the present value of one life was $1,324,446 in 2009 USD. Therefore, the present value loss of total deaths was 0.63 billion 2009 USD or 0.53 billion 2002 USD.

In sum, in 2002, the total costs for 35-44 individuals with asthma was estimated to be: 4.58 billion 2002 USD (3.81 billion + 0.24 billion + 0.53 billion). On average, the costs of asthma per person in age 35-44 individuals with asthma was estimated to be $2,349 (4.58 billion 2002 USD divided by 1.95 million).

**Step 3: Quantifying costs of adult-onset asthma in middle age due to intentional high school absenteeism**

In 1980, the total student enrollment between grade nine and twelve in 1980 was 13,616,000, which was reported in the historical summary of public elementary and secondary school statistics from National Center for Education Statistics (https://nces.ed.gov/programs/digest/d08/tables/dt08\_032.asp).

In our sample selection process, among 3,993 included cohort members who answered the asthma survey questions, we excluded 125 individuals who had asthma before age 18 in order to maximize the intentional (non-childhood-health related) school absences and control for the possibility that teenagers’ asthma would cause school absenteeism. This proportion was calculated as 3.13% (125/3993). Therefore, we applied this proportion to calculate the number of high school students in 1980: (1-3.13%) × 13,616,000=13,189,819. These were individuals who were in high school in the late 1970s, and their health in middle age was negatively impacted by intentional high school absenteeism.

Next, we calculated the expected costs in 2002 at different levels of chronic absenteeism (CA) incurred from high school approximately 20 plus years ago. Using the predicted probability of adult-onset asthma in middle age estimated from the both full- and sub- samples, we calculate the marginal/incremental probability going from a level of CA to the next level, which was shown in column (G) of S1 Table 1. Using these marginal/incremental probabilities, the 13,189,819 high school students in 1980, and $2,349 average asthma costs in age group 35-44 in 2022, we calculated the 2002 expected costs of adult-onset asthma in middle age that can be attributed to going from a level of CA to the next level, as shown in column (H) “Marginal/Incremental Asthma Costs”. Finally, accumulating these marginal/incremental asthma costs gave “Costs above Baseline” for each level of CA, as shown in column (I). For instance, based on the predicted probabilities using the full sample cohort, if all individuals had 4 years of CA when attending high school in 1980, the 2002 asthma cost of 4 years of high school CA would be $817,576,365. Based on the predicted probabilities using the sub-sample cohort, if all individuals had 4 years of CA when attending high school in 1980, the 2002 asthma cost of 4 years of high school CA would be $1,011,002,515.

**S1 Table 1. Quantify Costs of Adult-onset Asthma in Middle Age**

**at Specific Number of Years of Chronic Absenteeism**

| **Number of Years Having Chronic Absenteeism in High School** | **Predicted Probability of**  **Adult-onset Asthma in Middle Age** | **Marginal/ Incremental Probability** | **Marginal/ Incremental Asthma Costs** | **Costs above Baseline** |
| --- | --- | --- | --- | --- |
| **(E)** | **(F)** | **(G)** | **(H)^a^** | **(I)^b^** |
| **Full Sample** | | | | |
| 0 | 0.050 | Baseline | Baseline | Baseline |
| 1 | 0.056 | 0.006 | $175,084,282 | $175,084,282 |
| 2 | 0.062 | 0.006 | $193,519,099 | $368,603,381 |
| 3 | 0.069 | 0.007 | $213,596,008 | $582,199,389 |
| 4 | 0.077 | 0.008 | $235,376,976 | $817,576,365 |
| **Sub-Sample^c^** | | | | |
| 0 | 0.047 | Baseline | Baseline | Baseline |
| 1 | 0.054 | 0.007 | $208,174,003 | $208,174,003 |
| 2 | 0.062 | 0.008 | $235,779,754 | $443,953,757 |
| 3 | 0.070 | 0.009 | $266,514,775 | $710,468,532 |
| 4 | 0.080 | 0.010 | $300,533,983 | $1,011,002,515 |

^a^ Column (H) = High school students in 1980 (13,189,819) × Column (G). × Average asthma costs in age group

35-44 in 2002 ($2,349).

^b^ Column (I) is the cumulative costs of Column (H).

^c^ Our sub-sample excluded cohort members with overweight and obese BMI when they were young to control for

potentially high initial health risks.

1. **Sensitivity Analysis I**

In the sensitivity analysis I, we ran zero-inflated binomial regression models. The descriptive statistics of our samples showed that at least 93% of individuals not having adult-onset asthma in middle age, which indicated our outcome variable had a high proportion of zeros.

S1 Table 2 showed the estimation results using zero-inflated binomial regression models with number of years having CA as the key explanatory variable. The findings were consistent with those using the logistic regression models. Using the full sample, more years of CA in high school was associated with a higher risk of adult-onset asthma in middle age (OR = 1.12; 95% CI, 0.98-1.28). This association was marginally significant at the P=0.100 level as the P-value =0.103. Male had a lower risk of adult-onset asthma in middle age than female (OR = 0.31; 95% CI, 0.22-0.45). This was significant at the P=0.01 level. Individuals who never married or in other situations in their adulthood had a higher risk of adult-onset asthma in middle age than those who were married and spouse present (OR = 1.40; 95% CI, 0.99-1.96). This was marginally significant at the P=0.050 level. BMI in mid-adulthood was significant at the P-value =0.010 level. Hence, cohort members with normal/underweight BMI in mid-adulthood had a lower risk of adult-onset asthma than those who were overweight in mid-adulthood (OR = 0.52; 95% CI, 0.36-0.76). People who did not have bad habit before age 18 had a lower risk of adult-onset asthma in middle age than those who had bad habit (OR = 0.73; 95% CI, 0.53-1.01), which was significant at the P=0.100 level (P-value =0.057). If either one of biological parents had asthma, the individuals would have a significantly (P<0.100) higher risk of adult-onset asthma in middle age than those who did not report their parents having asthma (OR=2.17; 95% CI, 0.88-5.38).

The zero inflated binominal regression using sub-sample cohort confirmed findings of the full sample analysis. The association between years of CA in high school and adult-onset asthma in middle age was significant (P=0.057). Non-black non-Hispanic were less likely to have adult-onset asthma in middle age than Hispanic, which was significant at the P=0.100 level. Male had a lower risk of adult-onset asthma in middle age than female. This was significant at the P=0.010 level. Individuals who never married or in other situations in their mid-adulthood had a higher risk of adult-onset asthma in middle age than those who were married and spouse present. This was significant at the P=0.010 level. Cohort members with normal/underweight BMI in mid-adulthood were less likely to have adult-onset asthma than those who were overweight in mid-adulthood. This was significant at the P=0.010 level. People who did not have bad habit before age 18 had a lower risk of adult-onset asthma in middle age than those who had bad habit before 18, which was significant at the P=0.050 level as the P-value=0.029. One of parents had asthma is no longer significant in the sub-sample estimations. This implies that individuals who reported their parents having asthma were mostly those classified as overweight/obese at age between 20 and 22, who were excluded in the sub-sample.

**S1 Table 2. Zero Inflated Binominal Regression Model with Number of Years of Chronic Absenteeism as the Key Explanatory Variable**

| **Variable** | **Full Sample** | | | | | | | **Sub-Sample^c^** | | | | | | | |  |
| --- | --- | --- | --- | --- | --- | --- | --- | --- | --- | --- | --- | --- | --- | --- | --- | --- |
|  | **OR^a^** | **95% CI^b^** | | | | | **P-value** | **OR^a^** | **95% CI^b^** | | | | | **P-value** | |  |
| **Key Explanatory Variable** | | | | | | | | | | | | | | | |  |
| Number of Years Having Chronic Absenteeism | 1.12 | ( | 0.98 | , | 1.28 | ) | 0.103 | 1.15 | ( | 1.00 | , | 1.33 | ) | | 0.057 | |
| **Demographic Category (D)** | | | | | | | | | | | | | | | |  |
| Race/Ethnicity | | | | | | | | | | | | | | | |  |
| Black | 0.82 | ( | 0.49 | , | 1.37 | ) | 0.457 | 0.72 | ( | 0.42 | , | 1.24 | ) | | 0.232 | |
| Non-Black, Non-Hispanic | 0.67 | ( | 0.41 | , | 1.09 | ) | 0.107 | 0.64 | ( | 0.38 | , | 1.08 | ) | | 0.097 | |
| Hispanic | 1 |  |  |  |  |  |  | 1 |  |  |  |  |  | |  | |
| Gender | | | | | | | | | | | | | | | |  |
| Male | 0.31 | ( | 0.22 | , | 0.45 | ) | <0.010 | 0.32 | ( | 0.22 | , | 0.46 | ) | | <0.010 | |
| Female | 1 |  |  |  |  |  |  | 1 |  |  |  |  |  | |  | |
| Marital Status in Mid-adulthood | | | | | | | | | | | | | | | |  |
| Never Married/Other | 1.40 | ( | 0.99 | , | 1.96 | ) | 0.054 | 1.60 | ( | 1.12 | , | 2.30 | ) | | 0.010 | |
| Married, Spouse Present | 1 |  |  |  |  |  |  | 1 |  |  |  |  |  | |  | |
| Highest Grade Completed in Middle Age | | | | | | | | | | | | | | | |  |
| Less than Twelfth Grade | 1.19 | ( | 0.60 | , | 2.36 | ) | 0.612 | 0.85 | ( | 0.38 | , | 1.93 | ) | | 0.701 | |
| Twelfth Grade | 0.86 | ( | 0.56 | , | 1.32 | ) | 0.486 | 0.97 | ( | 0.62 | , | 1.53 | ) | | 0.907 | |
| Less than Four-Year College | 0.69 | ( | 0.43 | , | 1.11 | ) | 0.125 | 0.75 | ( | 0.45 | , | 1.24 | ) | | 0.265 | |
| At Least Four-Year College | 1 |  |  |  |  |  |  | 1 |  |  |  |  |  | |  | |
| **Economic Category (E)** | | | | | | | | | | | | | | | |  |
| Average Family Income Per Member During Adolescent/Young Adult | | | | | | | | | | | | | | | |  |
| < 33.3rd Percentile | 0.75 | ( | 0.48 | , | 1.17 | ) | 0.207 | 0.85 | ( | 0.53 | , | 1.37 | ) | | 0.511 | |
| Between 33.3rd and 66.7th Percentile | 0.93 | ( | 0.63 | , | 1.38 | ) | 0.717 | 0.96 | ( | 0.63 | , | 1.46 | ) | | 0.848 | |
| > 66.7th Percentile | 1 |  |  |  |  |  |  | 1 |  |  |  |  |  | |  | |
| Average Family Income Per Member in Mid-adulthood | | | | | | | | | | | | | | | |  |
| < 33.3rd Percentile | 0.92 | ( | 0.60 | , | 1.41 | ) | 0.692 | 0.72 | ( | 0.46 | , | 1.13 | ) | | 0.152 | |
| Between 33.3rd and 66.7th Percentile | 0.93 | ( | 0.62 | , | 1.40 | ) | 0.734 | 0.85 | ( | 0.56 | , | 1.31 | ) | | 0.462 | |
| > 66.7th Percentile | 1 |  |  |  |  |  |  | 1 |  |  |  |  |  | |  | |
| **Health Category (H)** | | | | | | | | | | | | | | | |  |
| BMI When Age in the 20, 21 and 22 | | | | | | | | | | | | | | | |  |
| Normal/Underweight | 1.01 | ( | 0.61 | , | 1.67 | ) | 0.962 | - |  |  | - |  |  | | - | |
| Overweight | 1 |  |  |  |  |  |  | - |  |  | - |  |  | | - | |
| BMI in Mid-adulthood | | | | | | | | | | | | | | | |  |
| Normal/Underweight | 0.52 | ( | 0.36 | , | 0.76 | ) | <0.010 | 0.50 | ( | 0.34 | , | 0.73 | ) | | <0.010 | |
| Overweight | 1 |  |  |  |  |  |  | 1 |  |  |  |  |  | |  | |
| Bad Habit When Age<18 | | | | | | | | | | | | | | | |  |
| No | 0.73 | ( | 0.53 | , | 1.01 | ) | 0.057 | 0.68 | ( | 0.48 | , | 0.96 | ) | | 0.029 | |
| Yes | 1 |  |  |  |  |  |  | 1 |  |  |  |  |  | |  | |
| Either One of Parents had Asthma | | | | | | | | | | | | | | | |  |
| Yes | 2.17 | ( | 0.88 | , | 5.38 | ) | 0.094 | 1.94 | ( | 0.72 | , | 5.18 | ) | | 0.189 | |
| No | 1 |  |  |  |  |  |  | 1 |  |  |  |  |  | |  | |

^a^ OR: Odd Ratios.

^b^ CI: Confidence Interval.

^c^ Our sub-sample excluded cohort members with overweight and obese BMI when they were young to control for potentially high initial health

risks.

1. **Sensitivity Analysis II**

Sensitivity analysis II was to change the threshold for defining CA from benchmark’s threshold of 15 days to a new threshold of 18 days. Now, an individual who missed more than 18 days in a year was considered as having CA in that year. We assigned a 1 or 0 indicator to each grade based on whether the cohort member had CA (new threshold) in that grade, then accumulated over the 4 years from grades nine to twelve as the measurement of number of years having CA (new threshold) for the cohort member. For both full sample and sub-sample, we ran regression models using the cumulative years having CA from grades nine to twelve as the key *explanatory* variable. For example, an individual *John* could have CA in grades nine and eleven, so *John*’s cumulative years of having CA is 2. Another individual *Mary* could have CA in grades ten and twelve, so *Mary*’s cumulative years of having CA is also 2.

For the full sample cohort, having a chronic disease *Y* at age 40 or in the mid-40s for each cohort member *i* was modeled as the following:

$$\mathrm{Logit} \left( Y_{i}=1 \right)=\beta_{0}+\beta_{1}\times\text{Number\_Years\_Chronic\_Absence}_{i}+B_{2}\text{×}D+B_{3}\times E+B_{4}\times H$$

$$=\beta_{0}+\beta_{1}\times{\text{Number}\text{\_Years\_Chronic\_Absence}}_{i}$$

$$+\beta_{2}\times\text{Race}_{i}+\beta_{3}\times\mathrm{Gender}_{i}+\beta_{4}\times{Marital\_Status\_40}_{i}$$

$$+\beta_{5}\times\text{Income\_20}_{i}+\beta_{6}\times\text{Income\_40}_{i}$$

$$+\beta_{7}\times\text{BMI\_20}_{i}+\beta_{8}\times\text{BMI\_40}_{i}+\beta_{9}\times\text{Bad\_Habit\_20}_{i}+\beta_{10}\times{\text{Parent}\text{\_}\text{Asthma}}_{i}$$

where *Y_i_*=1 was the outcome indicator for having adult-onset asthma at age 40 or in the mid-40s, *β_0_* was the intercept, Number_Years_Chronic_Absence_i_ was the cumulative years having CA from grades nine to twelve for a cohort member. *D* represented demographic variables, including Race_i_ indicating cohort member’s race/ethnicity, Gender_i_ indicating a cohort member’s gender, Marital_Status_40_i_ indicating marital status when the cohort member's age was between 38 to 42; *E* represented economic variables, including Income_20_i_ indicating the percentile classification of mean AFIPFMs when the cohort member’s age was between 14 and 26, Income_40_i_ indicating percentile classification of mean AFIPFMs when the cohort member’s age was between 38 to 42; *H* represents health variables, including BMI_20_i_ indicating the grouping for a cohort member’s mean BMIs when the individual was at age 20, 21 and 22, BMI_40_i_ indicating the grouping of mean BMIs when age was between 38 to 42, Bad_Habit_20_i_ indicating for bad habit, i.e. daily smoking or regular drinking in a week when cohort members were less than 18 years old, Parent_Asthma_i_ indicating one of the biological parents having asthma as one of the chronic health problems.

For the sub-sample cohort who were identified using the “Normal/Underweight” BMI group in BMI_20_i_, we also estimated the outcome having asthma at age 40 or in the mid-40s using the following explanatory variables:

$$\mathrm{Logit} \left( Y_{i}=1 \right)=\beta_{0}+\beta_{1}\times\text{Number\_Years\_Chronic\_Absence}_{i}+B_{2}\text{×}D+B_{3}\times E+B_{4}\times H$$

$$=\beta_{0}+\beta_{1}\times{\text{Number}\text{\_Years\_Chronic\_Absence}}_{i}$$

$$+\beta_{2}\times\text{Race}_{i}+\beta_{3}\times\mathrm{Gender}_{i}+\beta_{4}\times{Marital\_Status\_40}_{i}$$

$$+\beta_{5}\times\text{Income\_20}_{i}+\beta_{6}\times\text{Income\_40}_{i}$$

$$+\beta_{8}\times\text{BMI\_40}_{i}+\beta_{9}\times\text{Bad\_Habit\_20}_{i}+\beta_{10}\times{\text{Parent}\text{\_}\text{Asthma}}_{i}$$

where *Y_i_*=1 was the outcome indicator for having adult-onset asthma at age 40 or in the mid-40s, *β_0_* was the intercept, Number_Years_Chronic_Absence_i_ was the cumulative years having CA from grades nine to twelve for a cohort member. Because the sub-sample cohort was derived based on the variable BMI_20_i_, this variable was not included in the sub-sample estimation. The rest of variables in *D*, *E*, *H* categories were defined the same way as the previous paragraph.

Then we repeated the regression analyses using the new threshold. The results were shown in S1 Table 3 and S1 Table 4.

S1 Table 3 showed descriptive statistics using the new threshold. There were not any significant differences in all characteristics between the full sample and sub-sample. Nearly 40% of the cohort members in each sample had a least one year of CA (new threshold) between grades nine and twelve. This percentage is smaller than that in the results using the benchmark threshold of 15 days. Hence, the new threshold of 18 days is more stringent. More specifically, the proportion of cohort members having CA (new threshold) in grade nine was about 16%, grade ten was 18%, grade eleven was nearly 18%, and grade twelve was about 17%. These percentage points using the new threshold were lower than those using the benchmark.

**S1 Table 3. Descriptive Statistics of Chronic Absenteeism (New Threshold) in the Full Sample and Sub-Sample**

| **Characteristics** | **Full Sample** | | **Sub-Sample^a^** | | **P-Value^b^** |
| --- | --- | --- | --- | --- | --- |
|  | **(N=2626)** | | **(N=2372)** | |  |
|  | **n** | **%** | **n** | **%** |  |
| Number of Years Having Chronic Absenteeism | | | | | |
| 0 | 1515 | 57.7 | 1387 | 58.5 | 0.969 |
| 1 | 616 | 23.5 | 554 | 23.4 |  |
| 2 | 287 | 10.9 | 251 | 10.6 |  |
| 3 | 152 | 5.8 | 134 | 5.6 |  |
| 4 | 56 | 2.1 | 46 | 1.9 |  |
| Chronic Absenteeism in Grade Nine | | | | | |
| Yes | 443 | 16.9 | 379 | 16.0 | 0.396 |
| No | 2183 | 83.1 | 1993 | 84.0 |  |
| Chronic Absenteeism in Grade Ten | | | | | |
| Yes | 493 | 18.8 | 434 | 18.3 | 0.665 |
| No | 2133 | 81.2 | 1938 | 81.7 |  |
| Chronic Absenteeism in Grade Eleven | | | | | |
| Yes | 474 | 18.1 | 418 | 17.6 | 0.693 |
| No | 2152 | 81.9 | 1954 | 82.4 |  |
| Chronic Absenteeism in Grade Twelve | | | | | |
| Yes | 460 | 17.5 | 411 | 17.3 | 0.860 |
| No | 2166 | 82.5 | 1961 | 82.7 |  |

^a^ Our sub-sample excluded cohort members with overweight and obese BMI when they were young to control

for potentially high initial health risks.

^b^ Calculated by Chi-square test.

S1 Table 4 showed the results with number of years of CA (new threshold) as the key explanatory variable. The findings using the new threshold were consistent with those using the benchmark threshold of 15 days. Using the full sample, more years of CA (new threshold) in high school was associated with a higher risk of adult-onset asthma in middle age (OR = 1.13; 95% CI, 0.97-1.32). This association was marginally significant at the P=0.100 level as the P-value =0.105. Male had a lower risk of adult-onset asthma in middle age than female (OR = 0.31; 95% CI, 0.22-0.45). This was significant at the P=0.01 level. Individuals who never married or in other situations in their adulthood had a higher risk of adult-onset asthma in middle age than those who were married and spouse present (OR = 1.40; 95% CI, 1.00-1.97). This was significant at the P=0.050 level. BMI in mid-adulthood was significant (P-value =0.010). Hence, cohort members with normal/underweight BMI in mid-adulthood had a lower risk of adult-onset asthma than those who were overweight in mid-adulthood (OR = 0.52; 95% CI, 0.36-0.75). People who did not have bad habit before age 18 had a lower risk of adult-onset asthma in middle age than those who had bad habit (OR = 0.73; 95% CI, 0.53-1.01), which was significant at the P=0.100 level (P-value =0.058). If either one of biological parents had asthma, the individuals would have a significantly (P<0.10) higher risk of adult-onset asthma in middle age than those who did not report their parents having asthma (OR=2.19; 95% CI, 0.89-5.43).

The new threshold analysis using sub-sample cohort confirmed findings of the full sample (new threshold) analysis. All the notable associations that were found in the full sample (new threshold) analysis remained so in the sub-sample (new threshold) analysis. The association between years of CA (new threshold) in high school and adult-onset asthma in middle age was significant (P=0.058). Non-black non-Hispanic were less likely to have adult-onset asthma in middle age than Hispanic, which was significant at the P=0.100 level. Male had a lower risk of adult-onset asthma in middle age than female. This was significant at the P=0.010 level. Individuals who never married or in other situations in mid-adulthood had a higher risk of adult-onset asthma in middle age than those who were married and spouse present. This was significant at the P=0.010 level. Cohort members with normal/underweight BMI in mid-adulthood were less likely to have adult-onset asthma than those who were overweight in mid-adulthood. This was significant at the P=0.010 level. People who did not have bad habit before age 18 had a lower risk of adult-onset asthma in middle age than those who had bad habit before 18, which was significant at the P=0.050 level as the P-value=0.028. One of parents had asthma is not significant in the sub-sample (new threshold) estimations. This implies that individuals who reported their parents having asthma were mostly those classified as overweight/obese at age between 20 and 22, who were excluded in the sub-sample (new threshold).

**S1 Table 4. Logistic Regression Model with Number of Years of Chronic Absenteeism (New Threshold) as the Key Explanatory Variable**

| **Variable** | **Full Sample** | | | | | | | **Sub-Sample^c^** | | | | | | |
| --- | --- | --- | --- | --- | --- | --- | --- | --- | --- | --- | --- | --- | --- | --- |
|  | **OR^a^** | **95% CI^b^** | | | | | **P-value** | **OR^a^** | **95% CI^b^** | | | | | **P-value** |
| **Key Explanatory Variable** | | | | | | | | | | | | | | |
| Number of Years Having Chronic Absenteeism | 1.13 | ( | 0.97 | , | 1.32 | ) | 0.105 | 1.17 | ( | 1.00 | , | 1.37 | ) | 0.058 |
| **Demographic Category (D)** | | | | | | | | | | | | | | |
| Race/Ethnicity | | | | | | | | | | | | | | |
| Black | 0.82 | ( | 0.49 | , | 1.38 | ) | 0.459 | 0.72 | ( | 0.42 | , | 1.24 | ) | 0.232 |
| Non-Black, Non-Hispanic | 0.67 | ( | 0.41 | , | 1.09 | ) | 0.109 | 0.65 | ( | 0.38 | , | 1.09 | ) | 0.100 |
| Hispanic | 1 |  |  |  |  |  |  | 1 |  |  |  |  |  |  |
| Gender | | | | | | | | | | | | | | |
| Male | 0.31 | ( | 0.22 | , | 0.45 | ) | <0.010 | 0.31 | ( | 0.22 | , | 0.46 | ) | <0.010 |
| Female | 1 |  |  |  |  |  |  | 1 |  |  |  |  |  |  |
| Marital Status in Mid-adulthood | | | | | | | | | | | | | | |
| Never Married/Other | 1.40 | ( | 1.00 | , | 1.97 | ) | 0.050 | 1.61 | ( | 1.13 | , | 2.32 | ) | <0.010 |
| Married, Spouse Present | 1 |  |  |  |  |  |  | 1 |  |  |  |  |  |  |
| Highest Grade Completed in Middle Age | | | | | | | | | | | | | | |
| Less than Twelfth Grade | 1.17 | ( | 0.59 | , | 2.32 | ) | 0.652 | 0.83 | ( | 0.37 | , | 1.88 | ) | 0.654 |
| Twelfth Grade | 0.86 | ( | 0.56 | , | 1.33 | ) | 0.501 | 0.98 | ( | 0.62 | , | 1.54 | ) | 0.933 |
| Less than Four-Year College | 0.69 | ( | 0.43 | , | 1.11 | ) | 0.125 | 0.75 | ( | 0.45 | , | 1.24 | ) | 0.262 |
| At Least Four-Year College | 1 |  |  |  |  |  |  | 1 |  |  |  |  |  |  |
| **Economic Category (E)** | | | | | | | | | | | | | | |
| Average Family Income Per Member During Adolescent/Young Adult | | | | | | | | | | | | | | |
| < 33.3rd Percentile | 0.75 | ( | 0.48 | , | 1.18 | ) | 0.210 | 0.85 | ( | 0.53 | , | 1.37 | ) | 0.514 |
| Between 33.3rd and 66.7th Percentile | 0.93 | ( | 0.63 | , | 1.38 | ) | 0.709 | 0.96 | ( | 0.63 | , | 1.45 | ) | 0.834 |
| > 66.7th Percentile | 1 |  |  |  |  |  |  | 1 |  |  |  |  |  |  |
| Average Family Income Per Member in Mid-adulthood | | | | | | | | | | | | | | |
| < 33.3rd Percentile | 0.91 | ( | 0.60 | , | 1.40 | ) | 0.682 | 0.71 | ( | 0.45 | , | 1.12 | ) | 0.143 |
| Between 33.3rd and 66.7th Percentile | 0.93 | ( | 0.62 | , | 1.40 | ) | 0.740 | 0.86 | ( | 0.56 | , | 1.31 | ) | 0.471 |
| > 66.7th Percentile | 1 |  |  |  |  |  |  | 1 |  |  |  |  |  |  |
| **Health Category (H)** | | | | | | | | | | | | | | |
| BMI When Age in the 20, 21 and 22 | | | | | | | | | | | | | | |
| Normal/Underweight | 1.02 | ( | 0.62 | , | 1.68 | ) | 0.942 | - |  |  | - |  |  | - |
| Overweight | 1 |  |  |  |  |  |  | - |  |  | - |  |  | - |
| BMI in Mid-adulthood | | | | | | | | | | | | | | |
| Normal/Underweight | 0.52 | ( | 0.36 | , | 0.75 | ) | <0.010 | 0.50 | ( | 0.34 | , | 0.73 | ) | <0.010 |
| Overweight | 1 |  |  |  |  |  |  | 1 |  |  |  |  |  |  |
| Bad Habit When Age<18 | | | | | | | | | | | | | | |
| No | 0.73 | ( | 0.53 | , | 1.01 | ) | 0.058 | 0.68 | ( | 0.48 | , | 0.96 | ) | 0.028 |
| Yes | 1 |  |  |  |  |  |  | 1 |  |  |  |  |  |  |
| Either One of Parents had Asthma | | | | | | | | | | | | | | |
| Yes | 2.19 | ( | 0.89 | , | 5.43 | ) | 0.089 | 1.97 | ( | 0.74 | , | 5.26 | ) | 0.176 |
| No | 1 |  |  |  |  |  |  | 1 |  |  |  |  |  |  |

^a^ OR: Odd Ratios.

^b^ CI: Confidence Interval.

^c^ Our sub-sample excluded cohort members with overweight and obese BMI when they were young to control for potentially high initial health

risks.

**References**

1. Barnett SBL, Nurmagambetov TA. Costs of asthma in the United States: 2002-2007. Journal of allergy and clinical immunology. 2011;127(1):145-52.
